# Effects of maternal hepatitis B surface antigen positive status on the pregnancy outcomes: A retrospective study in Xiamen, China, 2011-2018

**DOI:** 10.1371/journal.pone.0229732

**Published:** 2020-03-10

**Authors:** Yan Zhao, Yin-ling Chen, Hai-qu Song, Pei-ying Huang, Li-ying Wang, Wei Liu, Bing-kun Huang, Fu-ping Lv, Caoxin Huang, Bing Yan, Xue-jun Li

**Affiliations:** 1 Xiamen Diabetes Institute, Xiamen, China; 2 Xiamen University Medical College, Xiamen, China; 3 Department of Endocrinology and Diabetes, the First Affiliated Hospital of Xiamen University, Xiamen, China; University of Mississippi Medical Center, UNITED STATES

## Abstract

**Objective:**

Hepatitis B virus infection is a major social and economic burden in developing countries, especially in China. We aimed to evaluate the effects of hepatitis B surface antigen (HBsAg) positive status on the pregnancy outcomes in the Chinese population.

**Methods:**

This retrospective cohort study was performed using data from the Medical Birth Registry of Xiamen, China, from January 2011 to March 2018. Multivariate logistic regression analysis was used to assess the association between the HBsAg status and pregnancy outcomes.

**Results:**

This study included 3,789 HBsAg-positive women and 29, 648 non-exposed women. The HBsAg-positive pregnant women were slightly older in age (29.3±4.3 vs. 28.9±4.4, *P*< 0.001). Additionally, pregnant women with a positive HBsAg status had higher odds of gestational diabetes mellitus (GDM) (adjusted odds ratio [aOR], 1.13; 95% confidence interval [CI], 1.03–1.23), and cesarean delivery (aOR, 1.12; 95%CI, 1.03–1.21). The risk of infants being large or small-for-gestational age, having low-birth weight, and of macrosomia, preterm birth, and stillbirth did not differ significantly between the HBsAg-positive and–negative women.

**Conclusion:**

In Xiamen, China, the slightly higher risk of GDM and cesarean section in women positive for HBsAg should not be neglected. Further studies should be conducted to evaluate the effects of HBsAg positivity on the pregnancy outcomes in different ethnic populations.

## Introduction

Hepatitis B virus infection is a significant public health problem, leading to social burden and high mortality worldwide [1]. It is reported that 240 million people are infected with the hepatitis B virus, which causes 600,000 deaths each year[2]. The largest burden of hepatitis B virus exists in China, where 74.6 million people are infected[3] and an estimated 4–10% of pregnant women are positive for hepatitis B surface antigen (HBsAg) [4].

Studies have reported that HBsAg positivity may be associated with pregnancy-induced hypertension, preeclampsia, fetal distress, and intrahepatic cholestasis[5,6].A subtle consequence of maternal HBsAg positive status is an increased mean infant birth weight[7]. High birth weight, especially macrosomia, is a determinant of obesity and high body mass index (BMI) in adolescence and adulthood[8,9]. In view of the increased risk for high birth weight, and subsequent obesity and the associated risk for diseases such as diabetes mellitus (DM), the negative health effects of HBsAg positivity during pregnancy are prolonged.

A study from China reported that pregnant women positive for HBsAg were at higher risk of gestational diabetes mellitus (GDM) and caesarean section.[5] A study from Hong Kong also reported that HBsAg positivity increased the risk of GDM with results specific to China[10]. These results, however, were not consistent with those of others studies.[11–13] In contrast, the association between HBsAg positivity status and GDM has not been confirmed in Asian and American pregnant women[4,13].Most published studies used data from countries where the prevalence of HBsAg positivity during pregnancy was low. Thus, the outcomes may not be applicable to higher risk countries and regions. Therefore, we performed this retrospective study to assess the association between HBsAg positivity and adverse pregnancy outcomes in the Chinese population.

## Materials and methods

### Population

This study included pregnant women with or without an HBsAg-positive status.All pregnant women were screened for hepatitis B virus infection by performing blood tests at their first prenatal visit. The data of both mothers and their offspring were collected from the Medical Birth Registry of Xiamen (MBRX) between January 2011 and March 2018. The MBRX was established in 2007 and is based on a compulsory notification of all live and stillbirths from 12 weeks’ gestation. The individual records of each woman and child are linked to the Xiamen citizen health information system using a unique identification number assigned to each Xiamen citizen. Every child is also linked with his or her biological mother’s identification number. All women are registered at their community health centers during each pregnancy and then referred to a secondary or tertiary hospital for healthcare from the 32nd gestational week until delivery. Offspring are given health examinations at birth (<3 days after birth); annual examinations are then performed until 3 years of age. The present study used only anonymized data from the MBRX database. No patients were involved as study participants.

### Inclusion and exclusion criteria

Only the records of women with no history of DM were included in the study. Pregnant women over 18 years of age underwent a 75-g oral glucose tolerance test (OGTT) between 24 and 28 weeks’ gestation for the diagnosis of GDM. The diagnostic criteria for GDM changed in China during the study period, as shown in Table 1. Therefore, for consistency, GDM diagnosis was based on the International Association of the Diabetes and Pregnancy Study Groups criteria. Pregnant women who had received medical treatment, such as oral glucocorticoids, thiazide diuretics, β-blockers, angiotensin-converting-enzyme inhibitors, or antiretroviral agents for chronic diseases were excluded. In addition, we excluded women with a fasting glucose level > 7.0 mmol/L before 12 weeks gestation who may have been diagnosed with diabetes before pregnancy.

**Table 1:** Diagnosis criteria of GDM from 2011 to 2018 in China.

IADPSG criteria	Chinese Ministry of Health (2011)	Guideline for T2DM in China (2013)	Guideline for GDM in China (2014-so far)
Fasting	5.1 mmol/L (92 mg/dl)	5.1 mmol/L (92 mg/dl)	5.1 mmol/L (92 mg/dl)
1h	10.0 mmol/L (180 mg/dl)	10.0 mmol/L (180 mg/dl)	10.0 mmol/L (180 mg/dl)
2h	8.5 mmol/L (153 mg/dl)	8.5 mmol/L (153 mg/dl)	8.5 mmol/L (153 mg/dl)
Diagnosis	Any abnormal blood glucose level can be diagnosed: one step or two steps (75 g OGTT or 50 g GCT to 75 g OGTT)	Any abnormal blood glucose level can be diagnosed: one step (75 g OGTT)	Any abnormal blood glucose level can be diagnosed: one step (75 g OGTT)

GDM, gestational diabetes mellitus; T2DM, type 2 diabetes mellitus; OGTT, oral glucose tolerance test.

### Data collection

The extracted information included: (1) maternal characteristics including: age, weight, height, BMI, obstetric history, education, family history of hypertension and DM, insulin treatment, antibiotic treatment, systolic blood pressure, diastolic blood pressure, fasting plasma glucose (FPG), parity, and results of OGTT; (2) birth outcomes: preterm birth, stillbirth, macrosomia, low-birth weight, large-for-gestational age (LGA) infant, small-for-gestational age (SGA) infant, cesarean delivery, and birth weight; and (3) delivery characteristics: GDM, HBsAg status, and gestational weight gain. For analysis, the BMI was calculated using weight (kg) divided by height (m) squared, and the women were divided into four groups based on the BMI (<18.5 kg/m^2^; 18.5–24.9 kg/m^2^; 25.0–27.9 kg/m^2^; and ≥ 28 kg/m^2^). The maternal age groups were as follows: < 25 years of age; 25–29 years of age; 30–34 years of age; 35–39 years of age; and≥ 40 years of age.[14]Parity was classified as 1 time and≥ 2 times. The level of maternal education was classified as > 9-year compulsory education and ≤ 9-year compulsory education. OGTT was performed at 3 time points (0 h, 1 h, and 2 h).

### Definition of pregnancy outcomes

The diagnostic criteria for GDM were based on the National Health and Family Planning Commission of the People’s Republic of China guidelines. When the 75 g OGTT results met or exceeded the following plasma glucose levels at the noted time-points, the women were diagnosed with GDM: 0 h, 5.1 mmol/L; 1 h, 10.0 mmol/L; and 2 h, 8.5 mmol/L. A 75 g OGTT was performed between the 24th and 28th weeks of gestation for all pregnant women who had not previously been diagnosed with diabetes. The test results were validated after 28 weeks. Macrosomia was diagnosed if birth weight was more than 4000g. LGA was ascertained by a birth weight more than the 90th percentile for the gestational age. SGA referred to birth weight less than the 10th percentile for the gestational age. The World Health Organization weight percentile calculator (3,542 ± 437g) was used to determine the weight percentile for babies born from 24 to 41 weeks’ gestation. Preterm birth was defined as birth before 37 weeks of pregnancy.

### Ethics statements

This retrospective study was approved by the ethics committee of human research of the First Affiliated Hospital of Xiamen University, and the requirement for obtaining informed consent was waived. The study was conducted in accordance with the tenets of the Declaration of Helsinki of 1975, revised in 2013, as well as, the relevant Chinese Good Clinical Practice regulations. The application number was KYH2018-007.

### Statistical analyses

Statistical analyses were performed using SPSS 18.0 (SPSS Inc, Chicago, IL, USA). All tests were two-tailed with significance set at *P*< 0.05. Additionally, multiple imputations were conducted for missing data in the multivariate analysis. Continuous variables were expressed as mean ±standard deviation (SD) and compared via the Student’s *t* test or one-way analysis of variance. Discontinuous variables were expressed as n (%) and compared using Pearson’s Chi-square (χ^2^) test. Multivariate logistic regression was used for multivariate analyses based on models containing factors to assess the associations among HBsAg positive status during pregnancy, GDM, and pregnancy outcomes. Some adjustment factors, age, BMI, and parity, affected the relation of HBsAg positivity status during pregnancy with GDM in Model 1. Several factors had effects on cesarean section in Model 2 included age, BMI, parity, insulin treatment, GDM, and antibiotic use. Factors in Model 1 or Model 2 could affect the pregnancy outcome. The dependent variable was the HBsAg status. GDM, LGA, SGA, macrosomia, low-birth weight, preterm birth, stillbirth, and cesarean delivery were the independent variables.

## Results

### Characteristics of pregnant women with or without HBsAg-positive status

Of the 33,437 pregnant women with data, 3,789 (11.3%) tested positive for HBsAg, and 29,648 (88.7%) tested negative (Table 2). The mean age of pregnant women in HBsAg positive group was greater than that of pregnant women in HBsAg negative group (29.3±4.3 vs. 28.9±4.4, *P*< 0.001). The proportion of study participants with an education level less than 9 years was higher in HBsAg positive group(28.0% vs. 25.0%), but the difference was not statistically significant (*P* = 0.054). The observed levels of FPG, OGTT, and blood pressure in the HBsAg positive group were slightly higher than those in the HBsAg negative group (all *P*< 0.05).

**Table 2:** The maternal characteristics of HBsAg-positive and–negative groups.

	HBsAg positive	HBsAg negative	*P* value
Numbers	3,789	29,648	
Age, years	29.3±4.3	28.9±4.4	< 0.001*
< 25, N (%)	389 (10.3)	3,969 (13.4)	< 0.001**
25–29, N (%)	1,780 (47.0)	14,216 (48.0)	
30–34, N (%)	1,117 (29.5)	7,765 (26.2)	
35–39, N (%)	401 (10.6)	2,939 (9.9)	
> 40, N (%)	75 (2.0)	537 (1.8)	
BMI, kg/m^2^	21.1±2.9	21.1±2.9	0.973
< 18.5, N (%)	681 (18.0)	5,389 (18.8)	0.967
18.5–24.9, N (%)	2,531 (66.8)	19,727 (66.5)	
25.0–27.9, N (%)	479 (12.6)	3,761 (12.7)	
> 28, N (%)	90 (2.4)	735 (2.5)	
Educations			
< 9 years, N (%)	1,062 (28.0)	7,869 (26.5)	0.054
≥ 9 years, N (%)	2,673 (70.6)	21,354 (72.0)	
Systolic blood pressure, mmHg	108.0±10.7	107.9±10.6	0.738
Diastolic blood pressure, mmHg	65.5±7.8	65.9±7.8	0.01*
Fasting plasma glucose, mmol/L	4.7±0.5	4.8±0.5	<0.001*
OGTT performed			
Fasting, mmol/L	4.5±0.4	4.5±0.4	0.952
1h, mmol/L	8.0±1.7	7.8±1.7	< 0.001*
2h, mmol/L	6.8±1.5	6.7±1.4	< 0.001*
Abnormal result, N (%)	757 (20.0)	5,263 (17.8)	< 0.001**
Family history of diabetes, N (%)	123 (3.3)	1,074 (3.6)	0.26
Family history of hypertension, N (%)	270 (7.1)	2,296 (7.7)	0.189
Parity			
1, N (%)	1,394 (36.8)	11,821 (39.9)	< 0.001**
≥ 2, N (%)	2,391 (63.1)	17,805 (60.1)	

Data showed as Mean±SD and N (%).

* indicates *P* derived from *t* test.

** indicates *P* derived from χ^2^ test.

### Association between the HBsAg status and pregnancy outcomes

The proportion of patients with GDM in both groups (20.0% vs. 17.8%) was the same as the prevalence of abnormal OGTT values(*P*< 0.001). Additionally, the proportion of cesarean section in the HBsAg positive group was higher (38.4%) than that in the HBsAg negative group (35.5%, *P* = 0.002). There was no statistically significant difference between the preterm birth rate in the HBsAg-positive and -negative group(6.1% vs. 5.6%, *P* = 0.195). The proportions of infants who were LGA, SGA, and of those who had macrosomia did not differ between the HBsAg-positive and -negative groups (all *P*> 0.05; Table 3).

**Table 3:** Pregnancy outcomes of mothers with HBsAG-positve and negative group.

	HBsAg positive n/N (%)	HBsAg negative n/N (%)	P*
GDM	757/3,789(20.0)	5,263/29,648(17.8)	< 0.001
LGA	564/3,122 (18.1)	4,137/24,192(17.1)	0.179
SGA	150/3,122 (4.8)	1,182/24,192 (4.9)	0.843
Macrosomia	99/3,117 (3.2)	746/24,172 (3.1)	0.785
Low-birth weight	173/3,117 (5.6)	1,397/24,172 (5.8)	0.605
Preterm birth	190/3,093 (6.1)	1,336/23,980 (5.6)	0.195
stillbirth	123/3,106 (4.0)	985/24,081 (4.1)	0.73
Cesarean section	1,188/3,095(38.4)	8517/23,980 (35.5)	0.002

Data showed as n/N (%).

*indicated P value derived from χ^2^ test. GDM, gestational diabetes mellitus; LGA, large-for-gestational age; SGA, small-for-gestational age.

### Effects of the HBsAg status on the pregnancy outcomes

Multivariate logistic regression analysis showed that the HBsAg-positive status was a slight risk factor for GDM (adjusted odds ratio [aOR], 1.13; 95% confidence interval [CI], 1.03–1.23; *P*< 0.001) after adjusting for variables including age, BMI, and parity. Furthermore, the HBsAg-positive status was also a slight risk factor for cesarean delivery (aOR, 1.12; 95% CI, 1.03–1.21; *P* = 0.011) after adjusting for age, BMI, parity, insulin use, GDM, and antibiotic use (Table 4).

**Table 4:** Effect of HBsAg positivity on pregnancy outcomes.

	Crude OR (95%CI)	Adjusted OR (95%CI)
GDM^a^	1.16 (1.06–1.26)	1.13 (1.03–1.23)
LGA	1.07 (0.97–1.178)	NS
SGA	0.98 (0.83–1.17)	NS
Macrosomia	1.03 (0.83–1.28)	NS
Low-birth weight	0.96(0.81–1.13)	NS
Preterm birth	1.11 (0.95–1.30)	NS
Stillbirth	0.97 (0.80–1.17)	NS
Cesarean section^b^	1.13 (1.05–1.22)	1.12 (1.03–1.21)

^a^Model 1, adjusted variables as following: age, BMI, and parity.

^b^ Model 2, adjusted variables included age, BMI, parity, insulin treatment, GDM, and antibiotic. GDM, gestational diabetes mellitus; LGA, large-for-gestational age; SGA, small-for-gestational age; BMI, body mass index; NS, no significance; OR, odd ratio; CI, confidence interval.

## Discussion

This study investigated the association between HBsAg status and pregnancy outcomes in China. We found that women with HBsAg positive status were tended to be slightly older than women with HBsAg negative status. This result is consistent with those of other studies, which also showed that women infected with the hepatitis B virus were more likely to be older [15–17].

Moreover, there is evidence that the number of abnormal blood glucose cases during pregnancy is higher among those with an HBsAg positive status than among those with an HBsAg-negative status. A large-sample cross-sectional study revealed that, when compared with patients who are HBsAg negative, HBsAg positive patients were more likely to develop DM[18]. Considering this evidence, it appears that hepatitis B virus infection may be a potential risk factor for DM. In the present study, the higher proportion of the HBsAg-positive women with abnormal blood glucose levels may be attributed to several factors. First, the liver plays a key role in regulating glucose homeostasis. Liver damage from the hepatitis B virus might cause a glycometabolic disorder[19], and an inflammatory condition might lead to defective glucose homeostasis. In addition, some studies have identified hepatitis B virus infection in the pancreas[20,21]. Hepatitis B virus replications in extrahepatic parts, like the pancreas, could also be responsible for causing DM and β-cell damage[21].Secondly, insulin resistance could also be associated with the pathogenesis of hepatogenous diabetes[22].

In our analysis, we found that pregnant women with an HBsAg positive status were at a slightly higher risk for preterm birth compared with HBsAg-negative women. Several large, cohort studies, have assessed the association between HBsAg-positive status and preterm birth[13,23]. Reddick and colleagues [13]reported that women with HBsAg-positive status had a higher risk of preterm birth than those with HBsAG-negative status, after adjusting for variables, such as age, insurance status, medical complications, and race. A study from Thailand also found that preterm birth was higher in women with HBeAg positive status[11]. However, one study did not identify an association between preterm birth and HBsAg positive status after adjusting for multiple variables[23]. Other studies performed in China also did not detect any relationship between the maternal HBsAg positive status and preterm birth[4,24]. These incompatible results might be due to differences in sample size, study design, and/or methods of collecting data. The reasons for preterm birth are multifactorial and intricate. The exact mechanism underlying preterm birth cannot be explained in most cases [25], but it is suspected that the accumulation of hepatitis B virus DNA initiates the placental inflammatory response and leads to preterm birth[11]. Further study is required to understand the mechanisms involved in viral infection and induction of preterm birth.

This study showed that an HBsAg positive status was a weak risk factor for GDM after adjusting for multiple variables. This may be because hepatitis B virus infection could cause insulin resistance, potentially via tumor necrosis factor alpha (TNF-α). Research indicates that TNF-αand TNF-receptor increase in patients with HBsAg positive status[26]. Serum ferritin concentration is also a risk factor for GDM in HBsAg positive individuals[17]. The increased serum ferritin level results in a higher risk of GDM, because exposure to HBsAg increases insulin resistance.

Our results regarding HBsAg positive status is a risk for caesarean delivery were not in line with those of previous studies[11,27]. Our study shows that women with HBsAg positive status during pregnancy are at a slightly higher risk for caesarean delivery. This may be due to concerns about infection during the birthing process. Although active and passive immunization is effective for preventing mother-to-infant transmission of hepatitis B virus, many clinicians may select caesarean delivery to reduce the risk of transmission. A high prevalence of caesarean delivery is noted in China for all pregnancies[28]. A study reported that the cesarean delivery rate in China was nearly 50% due to “social influence” rather than medical or obstetric indication.[29]

This is the first study to address the association between HBsAg positive status and pregnancy outcomes in Xiamen. In addition, we have included a relatively large sample size. However, there are some limitations of this study. Firstly, we included a large sample size, and the overall effect size was small. The risk of bias was not presented in the results. Secondly, these findings are from Xiamen, China; hence, they may only be applicable to the Chinese population. Thirdly, as this was a population-based cohort study, we were unable to collect data on all the associated and desired covariates from the MBRX database. For example, data on the HIV status, socioeconomic status, hepatitis C, and alcohol use were missing. Finally, this study lacked information regarding the hepatitis B e-antigen and liver function. The hepatitis B e-antigen is used as a marker for infectivity and its relevance during the immune clearance phase of hepatitis B virus infection. Therefore, further research should focus on the effect of hepatitis B e-antigen status on maternal outcomes; moreover, the future studies should include a longer follow-up period, greater number of variables, and larger sample sizes.

## Conclusions

Overall, in this retrospective study in China, the HBsAg-positive pregnant women were shown to have a slightly increased risk of GDM and cesarean delivery. Considering the higher risks, further studies should be conducted to evaluate the effects of HBsAg positivity on the pregnancy outcomes in different ethnic populations.
